# Beyond gait and balance: urinary and bowel dysfunction in X-linked adrenoleukodystrophy

**DOI:** 10.1186/s13023-020-01596-1

**Published:** 2021-01-06

**Authors:** Camille S. Corre, Natalie Grant, Reza Sadjadi, Douglas Hayden, Catherine Becker, Pablo Gomery, Florian S. Eichler

**Affiliations:** 1 Department of Neurology, Massachusetts General Hospital, 175 Cambridge Street, Suite 340, Boston, MA 02114 USA; 2 Harvard Medical School, Boston, MA USA; 3 Biostatistics Center, Massachusetts General Hospital, Boston, MA USA; 4 Department of Urology, Massachusetts General Hospital, Boston, MA USA

**Keywords:** Adrenoleukodystrophy, Adrenomyeloneuropathy, Urinary dysfunction, Bowel dysfunction, Urodynamics

## Abstract

**Objective:**

To characterize the prevalence, onset, and burden of urinary and bowel dysfunction in adult patients with adrenoleukodystrophy (ALD) and to evaluate any sex differences in symptom presentation.

**Methods:**

In this retrospective and prospective study, we performed medical record review (n = 103), analyzed the results of clinically indicated urodynamic testing (n = 11), and developed and distributed a symptom and quality of life (QOL) survey (n = 59).

**Results:**

Urinary and bowel symptoms are highly prevalent in both males (75.0%) and females (78.8%) in this population, most commonly urinary urgency, often leading to incontinence. Time to onset of first urinary or bowel symptom occurs approximately a decade earlier in males. Seventy-two percent of symptomatic patients report a limitation to QOL. Urodynamic evaluation provides evidence of three distinct mechanisms underlying lower urinary tract dysfunction: involuntary detrusor contractions (indicating uncontrolled neuronal stimulation with or without leakage), motor underactivity of the bladder, and asynergy between detrusor contraction and sphincter relaxation.

**Conclusions:**

Beyond gait and balance difficulties, urinary and bowel symptoms are common in adults with ALD and impair QOL. Males are affected at a younger age but both sexes experience a higher symptom burden with age. As this population also experiences gait and balance impairment, patients with ALD are more vulnerable to urinary urgency leading to incontinence. Urodynamic evaluation may help better elucidate the pathophysiologic mechanisms underlying neurogenic lower urinary tract dysfunction, which can allow more targeted treatment.

## Background

X-linked adrenoleukodystrophy (ALD) is a single gene disorder caused by mutations in the ABCD1 peroxisomal half-transporter gene, resulting in accumulation of very long chain fatty acids (VLCFAs) [1]. Clinical manifestations can be classified into two predominant phenotypes [2]. Cerebral adrenoleukodystrophy (CALD) is characterized by inflammatory demyelination in the brain and rapidly progresses to total neurological disability. Adrenomyeloneuropathy (AMN) presents in adulthood and affects the spinal cord and peripheral nerves, with a slower progression leading to gait and balance disturbances, sensory impairment, and bowel and bladder dysfunction [3]. Motor and sensory symptoms have been well-studied; less information is available on urinary and bowel dysfunction in adults with ALD.

Beyond a handful of early case reports and case series [4–10], two prospective studies have described the prevalence of urinary and bowel symptoms across larger cohorts. The first study (n = 46) assessed symptoms in females with ALD, finding both urinary and fecal incontinence to be common [11]. Notably, fecal incontinence was reported as an early symptom, even occurring in a few patients without signs of myelopathy on exam. The second study (n = 48) assessed symptoms in patients with AMN and reported that moderate to severe overactive bladder symptoms are highly prevalent, with symptoms being under-treated [12]. The majority of patients disclosed either no or “very minor” bowel symptoms. As these studies were limited by sex or phenotype, the prevalence and impact of these symptoms across the phenotypic spectrum have not been characterized to date.

To our best knowledge, there has not been a detailed study to systematically describe age of onset for urinary and bowel symptoms, urodynamic findings, symptom management, and the impact of symptoms on quality of life (QOL) in this patient population. Despite this gap in the literature, we have learned through clinical experience that both urinary and bowel symptoms can be a major source of disability in patients with ALD. We highlight this as an area of current unmet need and add to the literature with a combined retrospective and prospective study describing urinary and bowel symptoms in the largest cohort to date.

## Methods

We assessed the prevalence and severity of urinary and bowel symptoms in a population of adult patients with genetically or biochemically confirmed ALD through the following three methods: (1) retrospective medical record review, (2) survey development and distribution, and (3) urodynamic studies.

### Retrospective medical record review

We retrospectively reviewed electronic medical records of all adult patients with ALD seen in the Massachusetts General Hospital (MGH) Leukodystrophy Clinic by F.E. and C.B. between May 2005 and May 2019. We included patients with all ALD phenotypes in order to comprehensively assess the burden of urinary and bowel symptoms in ALD, particularly given the significant overlap of phenotypes among individuals. A rater (C.C.) systematically reviewed notes for all visits to this clinic, as well as any visits to the ALD Urology Clinic, where some patients were referred for evaluation by P.G. We recorded reports of the presence and age of onset of the following urinary and bowel symptoms: urinary frequency, urinary urgency, urinary incontinence, urinary hesitancy, urinary retention, fecal urgency, fecal incontinence, constipation, and diarrhea. We also documented medications or non-pharmacological interventions, plasma VLCFA levels, and concurrent neurological signs and symptoms.

In order to standardize our extraction of symptoms from medical records, we relied on definitions established by the International Continence Society (ICS) [13]. We documented the first chronological mention of the symptom as “age of onset”. The most recent age at which patients reported not having a symptom was also recorded for the purposes of computing age at symptom onset. When faced with unclear or ambiguous data, and to account for the insidious nature of the onset of these symptoms, we recorded a range over which the true value was reported to lie and included the full range in our analysis. The algorithm we developed to standardize ambiguous data is provided in Additional file 1.

### Survey development and distribution

We developed a brief survey (Additional file 2) designed to directly mirror the data collected through our retrospective medical record review. In developing this survey, we referenced the internationally-recognized symptom definitions established by the ICS and used their recommended language to phrase questions regarding symptom presence and age of onset. We asked patients to provide a medication list and surveyed the use of non-pharmacologic interventions and lifestyle modifications. To better understand the day-to-day realities of living with these symptoms, we asked about frequency of symptoms, estimated “warning time” between urgency and incontinence, and the patient’s own assessment of the impact of symptoms on QOL. Finally, we asked about the presence of other neurologic AMN symptoms, such as gait/balance impairment and neuropathy, and the use of any assistive mobility devices.

We distributed this survey to all adults with ALD seen in the Leukodystrophy Clinic at MGH. Additionally, we distributed the survey to a cohort of adults with ALD not seen as patients in our clinic, all family members of current patients. We used survey responses to support and supplement our retrospective medical record review. In the case of discrepancies between the two sources of data, we recorded the symptom as present if the patient endorsed it in at least one data source and we used the earliest reported age of onset. When evaluating survey responses regarding age of onset, we used the same set of rules developed for our retrospective medical record review.

### Urodynamic studies

We compiled and analyzed the results of all standard-of-care urodynamic studies (UDS) that patients in our cohort underwent in the MGH ALD Urology Clinic. These studies were performed by a urodynamicist always present for the study (P.G.) with the help of a technician, according to a standardized institutional protocol using a Laborie® system with a cystometrogram infusion rate between 10 and 30 mL/min.

The following procedures were performed:

- *Uroflowmetry,* in order to assess voiding function and to point towards the possibility of a poorly functioning bladder or bladder outlet obstruction (BOO).
- *Pressure-flow cystometrogram,* in order to simultaneously measure bladder pressure and voiding function. Detrusor pressure (P_det_) at peak urinary flow rate (Q_max_) is measured. P_det_@Q_max_ parameters are defined by the ICS pressure-flow nomogram [14]. Elevated values may indicate either dyssynergia between the detrusor and the urethral sphincter, or a bladder outlet/urethral obstruction [15]. Detrusor underactivity is defined by the ICS as P_det_@Q_max_ < 40 cm H_2_O and Q_max_ < 15 mL/s [14]. This study also assesses the volumes at which patients first become aware of bladder filling and the volume at which voiding becomes inevitable, with bladder filling at a controlled rate.
- *Abdominal pressure study and bladder pressure study,* to assess whether incontinence is likely to be caused by increased abdominal pressure (i.e. stress incontinence, leakage with Valsalva maneuver) versus increased bladder pressure from involuntary detrusor contractions. Detrusor contractions may or may not lead to incontinence. They may occur in phases or terminally.
- *Voiding cystourethrogram study with contrast,* to further assess the bladder wall as well as outlet obstruction. The vesical wall is observed to assess whether it appears smooth during filling and wide open during both filling and voiding.
- *EMG study,* to determine muscle depolarization and evaluate for any deviation from the normal relaxation of the external sphincter followed by sustained detrusor contraction.

### Data analysis

Statistical analysis was performed using SAS/STAT® University Edition 13.1. We first determined the cross-sectional prevalence of symptoms in our population. We then modeled the time to symptom onset using the ICLIFETEST procedure to perform a nonparametric survival analysis with interval-censored data [16, 17]. Ideally, our model would be based on a cumulative incidence function, which accounts for the competing risk of death prior to symptom onset, but we are not aware of any existing statistical program that is able to account for both competing risks and interval-censored data. We opted to include interval censoring rather than competing risks since our dataset relies so heavily on interval censoring (due to the recall bias of patient-reported data and the irregular timepoints inherent in retrospective data). The majority of our cohort was still living by the time of data analysis (all but six patients) and all six patients had a high symptom burden by the time of death, so we believe the effect of excluding competing risks in our model is negligible. We completed time to symptom onset analysis separately for males and for females, in order to determine any sex difference. We compared incidence between males and females using a generalized log-rank test with a chi-squared test with one degree of freedom. Finally, we ran a linear regression model to determine whether there was a correlation between C26:0 VLCFA levels and time to onset of urinary and bowel symptoms.

Two-sided p-values less than 0.05 were considered to be evidence of statistically significant effects. Since there was not a datapoint available for each symptom for every patient, we limited the population for the analysis of each symptom to patients for whom there was at least one recorded datapoint for that symptom.

## Results

### Study population

**Fig. 1:**
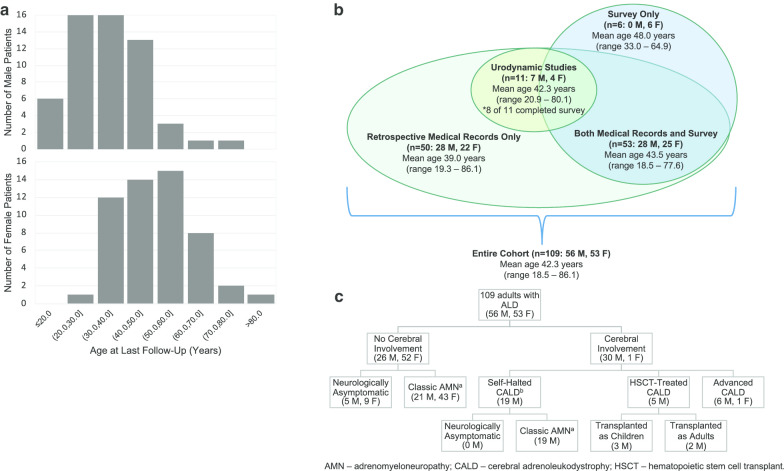
Descriptive statistics of the study population. Age distribution of males (top) and females (bottom) at last follow-up (**a**); Breakdown of study population into cohorts based on source of data (**b**); Phenotypic variability within our study population (**c**). ^a^Classic AMN defined as one or more of the following neurological signs and symptoms: gait disturbance, positive Romberg test, presence of foot drop, patient-reported frequent falls, use of one or more walking aids, or numbness/weakness/decreased vibration sensation in one or more extremities. We did not include patients presenting only with urinary and/or bowel symptom(s). ^b^Self-halted CALD defined as abnormal hyperintensity on brain MRI with no active gadolinium enhancement

We describe a population of 109 adults with ALD, of which 56 (51.4%) were male and 53 (48.6%) were female. Patients ranged from 18 to 86 years of age (mean 42.3 ± 14.7) at the time of most recent follow-up (Fig. 1a). Females ranged from 26 to 86 years of age (mean 49.9 ± 13.3); males ranged from 18 to 72 years of age (mean 35.2 ± 12.2). Retrospective medical record review was performed for 103 individuals, of which 16 (15.5%) had been seen in both the Leukodystrophy and ALD Urology Clinic. Of the 59 patients who completed surveys (57.3% response rate), six were not seen in either clinic. Cohort data are described in Fig. 1b. Of females in our cohort, 81.3% had a history of childbirth. Our population represents a spectrum of ALD phenotypes (Fig. 1c). At least one neurological sign or symptom (other than urinary and bowel symptoms) was found in 91.1% of males and 91.5% of females. Assistive mobility devices were used by 39.3% of males (10.7% unilateral, 3.6% bilateral, 25.0% wheelchair) and 25.5% of females (12.8% unilateral, 6.4% bilateral, 6.4% wheelchair).

### Cross-sectional symptom prevalence and age of onset

**Fig. 2:**
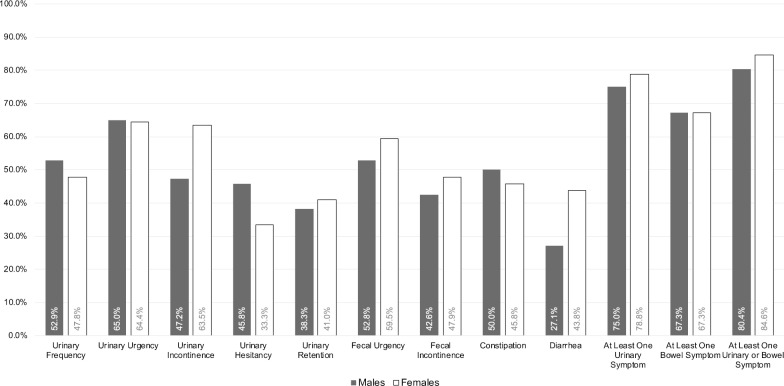
Symptom prevalence. Cross-sectional prevalence of each urinary and bowel symptom in our study population. Of note, mean age of females is approximately 15 years older than males; cross-sectional prevalence is not directly comparable between males and females

By the time of most recent follow-up, 76.9% of patients in our cohort (75.0% of males and 78.8% of females) had experienced at least one urinary symptom, and 67.3% had experienced at least one bowel symptom (same for both males and females). Among both males and females, the most common symptom was urinary urgency, followed in men by urinary frequency and fecal urgency and in women by urinary incontinence and fecal urgency. The cross-sectional prevalence of each symptom in our cohort is depicted in Fig. 2.

**Fig. 3:**
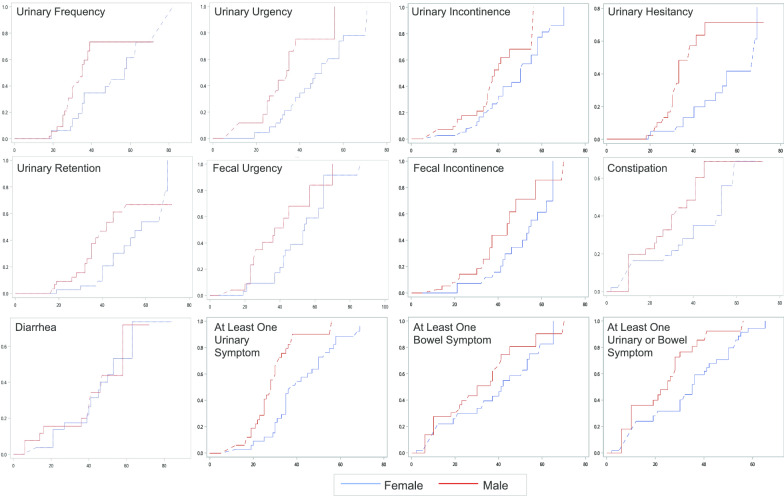
Time to symptom onset for males and females. Interval-censored models of time to symptom onset for males and females in our population. X-axis represents age in years and y-axis represents proportion of population who has developed symptom by a given age

Despite comparable cross-sectional symptom prevalence between males and females, several symptoms showed an earlier onset in males compared with females. This sex difference in time to symptom onset was statistically significant for each urinary symptom, as well as for fecal urgency (Table 1). There were no significant differences in time to onset of fecal incontinence, diarrhea, or constipation between males and females. In both sexes, urinary urgency was an early symptom (median time to onset 34 years for males and 50 for females). In males this was followed by urinary frequency (35 years) and fecal urgency (37 years). In females the onset of urinary urgency occurred at approximately the same age as urinary incontinence (50 years). When considering the onset of at least one urinary symptom, or at least one bowel symptom, symptoms appeared approximately a decade later in females compared with males. Models estimating population-wide time to symptom onset are presented in Fig. 3. There was no correlation between plasma VLCFA level (C26:0) and time to onset of symptoms. Other symptoms of myelopathy, such as gait/balance impairment, lower extremity weakness, or sensory abnormalities, also showed an earlier onset in males compared with females (median time to onset 30 years for males and 43 years for females). Of symptomatic patients in our cohort, 57.8% (53.6% of males and 62.3% of females) developed urinary or bowel symptoms prior to other symptoms of myelopathy.

**Table 1:** Onset age for each urinary and bowel symptom and myelopathy symptoms among males and females

	Males		Females		Equality of distributions
	n	Median (95% CI)	n	Median (95% CI)	Chi-squared (*p* value)
Urinary frequency	51	35 (30; 38)	46	57 (36; 63)	7.623 (0.006)**
Urinary urgency	40	34 (26; 35)	45	50 (40; 58)	10.672 (0.001)**
Urinary incontinence	53	38 (35; 45)	52	50 (42; 55)	4.953 (0.026)*
Urinary hesitancy	48	38 (32; 45)	45	68 (55; 69)	12.092 (< 0.001)**
Urinary retention	47	42 (35; –^a^)	39	58 (50; 69)	5.018 (0.025)*
Fecal urgency	36	37 (26; 45)	37	54 (43; 62)	4.913 (0.027)*
Fecal incontinence	47	44 (36; 48)	48	54 (46; 62)	3.759 (0.053)
Constipation	50	41 (30; 45)	48	53 (40; 59)	3.468 (0.063)
Diarrhea	48	58 (47; 58)	48	53 (45; 63)	0.037 (0.848)
At least one urinary symptom	56	28 (25; 30)	52	38 (35; 47)	17.937 (< 0.001)**
At least one bowel symptom	52	30 (22; 40)	52	41 (33; 53)	2.391 (0.122)
At least one urinary or bowel symptom	56	25 (10; 28)	52	35 (30; 41)	9.096 (0.003)**
Myelopathy	56	30 (28; 32)	53	43 (38; 48)	26.158 (< 0.001)**

Median point estimate of onset age in years for each urinary and bowel symptom and myelopathy symptoms, using generalized log-rank test to calculate equality distributions between males and females

^a^Model unable to compute value due to limited sample size within extremes of age distribution

^*^p < .05

^**^p < .01

### Impact on quality of life

**Fig. 4:**
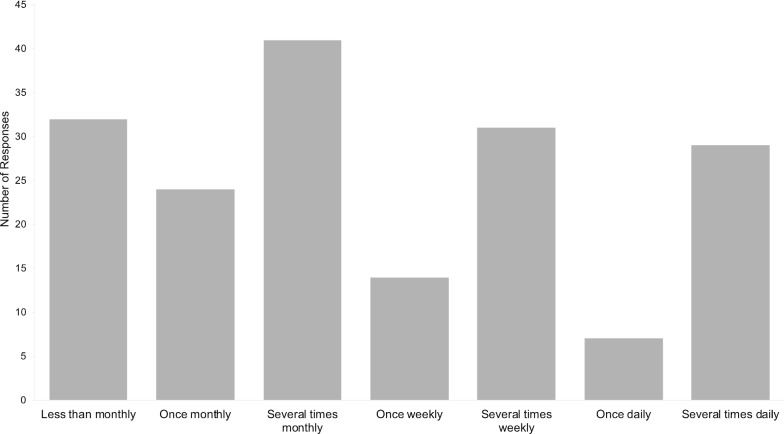
Frequency of urinary and bowel symptoms. Number of survey responses in each frequency range among all patient-symptom datapoints regarding frequency of urinary and bowel symptoms (n = 178)

When specifically questioned about QOL, 71.7% of our survey respondents who reported at least one urinary or bowel symptom also reported a limitation to QOL, and 39.6% reported a moderate or severe limitation to QOL. Among survey respondents who reported at least one urinary or bowel symptom, the frequency with which symptoms were experienced ranged from less than monthly to several times daily, with no apparent skew to either extreme (Fig. 4). Among patients who experience urinary frequency, the mean number of times they reported voiding in a 24-h period was 10.2 (95% CI: 8.3–12.1). Patients who had experienced urinary urgency that caused incontinence reported a mean “warning time” from urgency to inevitable incontinence of 3.8 min (95% CI: 1.7–5.8). Patients who had experienced fecal urgency that caused incontinence reported a mean “warning time” of 3.2 min (95% CI 2.1–4.3).

### Urodynamic studies

Standard-of-care UDS were performed at MGH on 11 (seven males and four females) of the symptomatic patients in our cohort, mean age 42.3 ± 18.4 years. One patient had a repeat study due to worsening urinary incontinence; the results of his initial, normal study were excluded in analysis. Descriptive statistics summarizing UDS findings are found in Table 2.

**Table 2:** Urodynamic data

ID	Phenotype	Sex	Age (yrs)	First sensation (mL)	Maximum cystometric capacity (mL)	Bladder pressure study	P_det_@Q_max_ (cm H_2_O)	Q_max_ (mL/s)	EMG finding	Vesical wall	Impression
1	AMN	F	63.6	"Very early in filling phase"	Normal	–	14	10	–	smooth	Neurogenic hypocontractile detrusor
2	AMN	F	80.2	112	263	Phasic detrusor contractions	56	11	Synergy	Smooth	NDO
3	AMN	M	57.4	155	155	No phasic detrusor contractions	13	5	Synergy	Markedly trabeculated	NDO, impaired contractility
4A^a^	CALD post-HSCT	M	21.9	195	411	No phasic detrusor contractions	42	19	Synergy	Smooth	Normal
4B^a^	CALD post-HSCT	M	23.2	62	139	Phasic detrusor contractions	26	11	Sphincter dyssynergia	–	NTDO with DSD
5	AMN, self-halted CALD	M	28.2	32	216	Phasic detrusor contractions	66	9	Synergy	Smooth	NDO, BOO, multi-phasic micturition
6	AMN, self-halted CALD	M	36.3	30–45	43	Terminal detrusor overactivity	62	3	Sphincter dyssynergia	Moderately trabeculated	NTDO with DSD
7	AMN, self-halted CALD	M	20.9	262	348	No phasic detrusor contractions	47	15	Synergy	Smooth	NTDO, neurogenic delay in sensory arm
8	AMN	F	47.2	130	431	Phasic detrusor contractions	8	3	Synergy	Smooth	NDO
9	AMN	F	47.2	132	545	Phasic detrusor contractions	20	10	Synergy	Smooth	NDO, impaired contractility
10	AMN	M	46.9	78	293	Phasic detrusor contractions	56	11	Synergy	Smooth	severe NDO
11	AMN, self-halted CALD	M	34.8	286	359	Phasic detrusor contractions	51	11	Synergy	Smooth	NDO

Summary of urodynamic patient data collected at Massachusetts General Hospital, describing a diverse array of neurogenic bladder presentations

^a^Patient 4 had urodynamic studies performed at two timepoints

BOO, bladder outlet obstruction; DSD, detrusor sphincter dyssynergia; HSCT, hematopoietic stem cell transplant; NDO, neurogenic detrusor overactivity; NTDO, neurogenic terminal detrusor overactivity; P_det_, detrusor pressure; Q_max_, peak urinary flow rate

Ten of 11 patients had findings suggestive of involuntary detrusor contractions during the filling or voiding phases, compared with the single sustained detrusor contraction that is characteristic of normal voiding. Each of these patients was determined to have neurogenic detrusor overactivity (NDO), which causes disproportionately large increases in vesical pressure as the bladder is filled. Cystometrogram also revealed phasic detrusor contractions in seven patients, as well as neurogenic terminal detrusor overactivity (NTDO) in one patient. Only one of 11 patients maintained normal compliance throughout the study, and four patients became incontinent immediately at (or within 10 mL of) their first sensation during the filling phase. No patients showed involuntary leakage with the Valsalva maneuver.

Other patients had a urodynamic presentation suggestive of motor underactivity of the bladder, which describes a primary defect of the voiding rather than filling phase. Of the five patients who met the ICS criteria for detrusor underactivity, three showed impaired contractility, one of whom additionally had an elevated maximum cystometric capacity (545 mL).

Finally, some patients’ findings suggest a neurogenic defect in synchrony between contraction of the detrusor muscle and relaxation of the urethral sphincter. Two patients had evident detrusor-sphincter dyssynergia (DSD), one of whom also had an abnormally high detrusor pressure at maximal flow. Interestingly, patient #5, though not found to have DSD, had a pressure-flow study which revealed BOO, indicating a high voiding detrusor pressure that does not yield a proportionally increased urine flow rate.

### Medications and compensation strategies

Patients use a variety of strategies to cope with these symptoms in their everyday lives. In our combined retrospective and prospective cohort, 53.4% of symptomatic patients had tried at least one medication for symptom management, and 70.8% had tried at least one lifestyle modification or other non-pharmacological intervention (Table 3). Among pharmacological interventions, laxatives and bowel supplements were the most commonly used (42.6%), followed by anticholinergics (19.7%), β3-agonists (12.7%), and α-blockers (11.3%). Only one patient reported the use of a cholinergic agonist. Of non-pharmacological interventions and lifestyle modifications, the most commonly reported methods were use of leak protection pads (41.6%), restriction of caffeine and/or alcohol intake (40.4%), and limitation or modification of fluid intake (37.1%).

**Table 3:** Methods for management of urinary and bowel symptoms

Pharmacological interventions^a^			Non-pharmacological interventions		Lifestyle modifications	
Constipation/bowel supplements^c^	Fiber supplements Laxatives Linaclotide Loperamide Lubiprostone Probiotics	42.6%	Leak protection pads/briefs^e^	41.6%	Restricting caffeine and/or alcohol intake^b,^^e^	40.4%
Anti-cholinergics^d^	Darifenacin Fesoterodine Oxybutynin Solifenacin Tolterodine Trospium	19.7%	Catheterization^d^	13.3%	Limiting/modifying fluid intake^e^	37.1%
β3-agonists^d^	Mirabegron	12.7%	Abdominal massage^b^^,e^	9.6%	Limiting/modifying food intake^e^	23.6%
α-blockers^d^	Alfuzosin Silodosin Tamsulosin Terazosin	11.3%	Pelvic floor physical therapy^e^	7.9%	Planning daily schedule around proximity to restroom^b,^^e^	17.3%
Cholinergic agonists^d^	Bethanechol	1.4%				

Percentage of patients in our cohort that had tried each medication, non-pharmacological intervention, and lifestyle modification to manage urinary and bowel symptoms

^a^Among patients with a medication record available

^b^Among survey respondents only

^c^Among patients with at least one bowel symptom

^d^Among patients with at least one urinary symptom

^e^Among patients with at least one urinary or bowel symptom

## Discussion

Mutations in ABCD1 with subsequent elevations in VLCFAs cause the most frequent metabolic hereditary spastic paraplegia. While it is known that this pathology results in a slowly progressive gait disorder due to spastic paraparesis and sensory ataxia, other symptoms remain poorly explored. We present here the largest study to date of urinary and bowel symptoms in adult patients with ALD. Our findings suggest that urinary and bowel symptoms are common in adults with ALD and limit quality of life for the majority of patients.

Onset of these symptoms occurs approximately a decade earlier in males than in females and may present more variably in females. In the majority of patients, urinary and bowel symptoms present prior to other symptoms of myelopathy. Despite an overlap in clinical symptomatology, urodynamic evaluation provides evidence of distinct pathophysiologic mechanisms underlying these symptoms, which can help guide treatment considerations. Based on our patients’ UDS findings, we describe three putative mechanisms for the urinary dysfunction seen in the adult ALD population: involuntary detrusor contractions (primarily during the filling phase), motor underactivity of the bladder (primarily during the voiding phase), and asynchrony between detrusor contraction and sphincter relaxation. The most common of these mechanisms is involuntary detrusor contractions, indicating uncontrolled neuronal stimulation with or without leakage. Notably, no patients in our cohort showed evidence of Valsalva leak points, indicating that the incontinence experienced by patients with ALD is likely distinct from the stress incontinence caused by increased abdominal pressure that may occur in healthy aging adults.

Urinary urgency is a common early symptom, often preceding incontinence. Particularly striking is our finding of extremely short “warning times” that patients reported from the first sensation of urinary or fecal urgency to involuntary urination or defecation (mean 3.8 and 3.2 min, respectively). Previous research has demonstrated that patients with ALD with impaired ambulation experience more severe urinary symptoms than patients with unaffected walking [18]; thus it is likely that patients who experience urgency and incontinence would be unable to reach a restroom in time. Most patients with gait difficulties and imbalance also endorsed both urinary and fecal urgency and incontinence, further compounding the poor QOL.

The common mechanism of involuntary detrusor contractions (10/11 patients) may play a key role in the presentation of urinary urgency and incontinence experienced by patients in our cohort. As fluid volume increases during bladder filling, these contractions cause disproportionately large increases in bladder pressure, interfering with the bladder’s ability to accommodate increasing volumes of urine without leakage. Specifically, 10 patients presented with NDO, seven with phasic contractions, and one with NTDO. Involuntary contractions may also be implicated in the urinary incontinence observed in 10 of the 11 patients over the course of the UDS.

Although less common, motor underactivity of the bladder during voiding, such as impaired contractility, impairs bladder emptying in some patients. The inability to adequately contract the bladder with effort contributes to a low urine flow rate and may manifest as urinary retention and/or hesitancy. In a third subset of patients, a defect in synchrony between detrusor contraction and sphincter relaxation may underly serious neuropathology. Notably, multiple mechanisms may be at play in a given patient.

These distinct mechanisms indicate that the common pathology of long tract degeneration along the spinal cord in AMN may produce overlapping constellations of symptoms in different patients. Consistent with previous studies, measurements of VLCFAs in plasma do not correlate with onset of symptoms. We suggest that UDS may be used to determine the mechanism of urinary dysfunction in a particular patient, rather than managing symptoms based on clinical presentation alone, so that treatment can appropriately target the root cause of symptoms. It may be appropriate to obtain a baseline UDS in young adult patients with ALD to track changes over time, and to repeat UDS with changes in clinical presentation.

Only one of the 11 patients had repeat UDS, the first being a normal functional study. Interestingly, he had symptomatic cerebral disease at the time of UDS. He had undergone allogeneic hematopoietic stem cell transplantation (HSCT) approximately two and a half years prior, which halted the inflammatory progression of his brain lesion but left him with severe neurological deficits. Combined with his relatively young age, it is possible that, at that time, the patient’s incontinence may have been attributable to CALD rather than AMN. Previous studies showed an association between rapid progression of urinary symptoms and active demyelinating cerebral disease [9] and reported deterioration of bladder function as a complication during transplant period [10].

Many of our findings mirror observations in other rare, phenotypically similar neurodegenerative conditions such as Friedreich’s Ataxia (FRDA) and hereditary spastic paraplegia (HSP). Similar to our finding that one or more urinary symptoms were present in the majority of patients, a large proportion of patients with HSP and FRDA report at least one urinary and bowel symptom [19, 20]. Females with HSP had higher rates of urinary frequency than males, which differs from our cohort, likely due to the X-linked nature of ALD. Similar to our ALD population, HSP patients on medication continued to complain of symptoms, indicating that these symptoms are both under-treated and that currently available medications may fail to provide adequate symptom relief.

There are a few limitations to our study that impact generalizability of our findings. First, given that the majority of our cohort comes from a patient population seeking neurological evaluation at an academic medical center, our data may overestimate the true prevalence in the broader ALD population. This potential bias may be at least partially mitigated by under-reporting from patients due to the sensitive nature of these symptoms and social stigma around discussing them. Second, urinary and bowel dysfunction are also prevalent in adult patients without ALD [21–25] and there are a number of factors that may contribute to the accumulation of symptoms with age (most notably, parity, diet, obesity, prostatic hypertrophy, surgical history, and concomitant medication use). Importantly, there may be more confounding factors, such as history of childbirth [26], contributing to females having a higher incidence of these symptoms in general. Third, our study is limited by sample size; though ALD is a rare disease and we present the largest cohort to date, we acknowledge that our findings, in particular our discussion of urodynamic patterns, are based on a limited number of patients and should be considered in the context of all available clinical information. Finally, the onset of urinary and bowel symptoms in ALD is an insidious process, and efforts to quantify prevalence and age of onset rely on heterogeneous and retrospective patient-reported data. While we made efforts to standardize and find commonalities among an array of patient stories, we recognize that the onset of these symptoms is highly individualized and may best be represented by a spectrum.

## Conclusion

Both males and females with ALD experience urinary and bowel symptoms that reduce quality of life, though the onset of first symptom occurs approximately a decade earlier in males compared with females. Concurrent gait and balance impairment may contribute to increased disease burden with symptoms of urgency. UDS can provide physiologic context for clinical presentations and may be beneficial in order to target symptoms with appropriate first-line treatment and to monitor changes over time.

## Supplementary information

**Additional file 1.** Standardized algorithm. Standardized approximations of the upper and lower bounds of the range for age of symptom onset.

**Additional file 2.** Urinary and bowel symptoms in ALD/AMN. Survey distributed to all adults with ALD seen in the MGH Leukodystrophy Clinic as well as a cohort of adults with ALD not seen as patients in our clinic.

## Data Availability

The datasets used and analyzed during the current study are available from the corresponding author on reasonable request.

## Abbreviations

ALD: X-linked adrenoleukodystrophy
AMN: Adrenomyeloneuropathy
BOO: Bladder outlet obstruction
CALD: Cerebral adrenoleukodystrophy
DSD: Detrusor-sphincter dyssynergia
FRDA: Friedreich’s Ataxia
HSCT: Hematopoietic stem cell transplantation
HSP: Hereditary spastic paraplegia
ICS: International Continence Society
MGH: Massachusetts General Hospital
NDO: Neurogenic detrusor overactivity
NTDO: Neurogenic terminal detrusor overactivity
P_det_: Detrusor pressure
Q_max_: Maximum urine flow rate
QOL: Quality of life
UDS: Urodynamic study
VLCFA: Very long chain fatty acid

## References

[1] Moser HW, Moser AB, Frayer KK (1981). Adrenoleukodystrophy: increased plasma content of saturated very long chain fatty acids. Neurology.

[2] Kemp S, Berger J, Aubourg P (2012). X-linked adrenoleukodystrophy: clinical, metabolic, genetic, and pathophysiological aspects. BiochimBiophysActa.

[3] Engelen M, Kemp S, de Visser M (2012). X-linked adrenoleukodystrophy (X-ALD): clinical presentation and guidelines for diagnosis, follow-up and management. Orphanet J Rare Dis.

[4] Shinbo H, Kageyama S, Hayami S (2001). Voiding dysfunction in a patient with adolescent adrenoleukodystrophy. Int J Urol.

[5] Walther MM, Cutler GB (1997). Urodynamic abnormalities in two brothers with adrenomyeloneuropathy. World J Urol.

[6] Sakakibara R, Hattori T, Fukutake T, Mori M, Yamanishi T, Yasuda K (1998). Micturitional disturbance in a patient with adrenomyeloneuropathy (AMN). NeurourolUrodyn.

[7] O’Neill BP, Mannion LC, Feringa ER (1981). Theadrenoleukomyeloneuropathy complex: expression in four generations. Neurology.

[8] Griffin JW, Goren E, Schaumburg H, Engel WK, Loriaux L (1977). Adrenomyeloneuropathy: a probable variant of adrenoleukodystrophy. Neurology.

[9] Silveri M, De Gennaro M, Gatti C, Bizzarri C, Mosiello G, Cappa M (2004). Voiding dysfunction in x-linked adrenoleukodystrophy: symptom score and urodynamic findings. J Urol.

[10] Kühl J-S, Suarez F, Gillett GT (2017). Long-term outcomes of allogeneic haematopoietic stem cell transplantation for adult cerebral X-linked adrenoleukodystrophy. Brain.

[11] Engelen M, Barbier M, Dijkstra IM (2014). X-linked adrenoleukodystrophy in women: a cross-sectional cohort study. Brain.

[12] Hofereiter J, Smith MD, Seth J (2015). Bladder and bowel dysfunction is common in both men and women with mutation of the ABCD1 gene for x-linked adrenoleukodystrophy. JIMD Rep.

[13] Haylen B. (ed.) ICS glossary of terminology. https://www.ics.org/glossary. Accessed 21 April 2020.

[14] Griffiths D, Höfner K, van Mastrigt R, Rollema HJ, Spångberg A, Gleason D (1997). Standardization of terminology of lower urinary tract function: pressure-flow studies of voiding, urethral resistance, and urethral obstruction. NeurourolUrodyn.

[15] Nitti VW (2005). Pressure flow urodynamics: the gold standard for diagnosing bladder outlet obstruction. Rev Urol.

[16] Wellner JA, Zhan Y (1997). A hybrid algorithm for computation of the nonparametric maximum likelihood estimator from censored data. J Am Stat Assoc.

[17] Guo C, So Y, Johnston G. Analyzing interval-censored data with ICLIFETEST procedure. In: SAS Institute Inc, 2014. https://support.sas.com/resources/papers/proceedings14/SAS279-2014.pdf. Accessed 21 April 2020.

[18] Huffnagel IC, van Ballegoij WJC, van Geel BM, Vos JMBW, Kemp S, Engelen M (2019). Progression of myelopathy in males with adrenoleukodystrophy: towards clinical trial readiness. Brain.

[19] Lad M, Parkinson MH, Rai M (2017). Urinary, bowel and sexual symptoms in a cohort of patients with Friedreich’s ataxia. Orphanet J Rare Dis.

[20] Braschinsky M, Zopp I, Kals M, Haldre S, Gross-Paju K (2010). Bladder dysfunction in hereditary spastic paraplegia: what to expect?. J NeurolNeurosurgPsychiatr.

[21] Coyne KS, Sexton CC, Bell JA (2013). The prevalence of lower urinary tract symptoms (LUTS) and overactive bladder (OAB) by racial/ethnic group and age: results from OAB-POLL. NeurourolUrodyn.

[22] Tennstedt SL, Link CL, Steers WD, McKinlay JB (2008). Prevalence of and risk factors for urine leakage in a racially and ethnically diverse population of adults: The Boston Area Community Health (BACH) survey. Am J Epidemiol.

[23] Ivo D, Devaki P, Luma HN (2014). Prevalence, trends, and risk factors for fecal incontinence in United States Adults, 2005–2010. ClinGastroenterolHepatol.

[24] Schiller LR, Pardi DS, Spiller R (2014). Gastro 2013 APDW/WCOG Shanghai working party report: chronic diarrhea: definition, classification, diagnosis. J GastroenterolHepatol.

[25] Stewart WF, Liberman JN, Sandler RS (1999). Epidemiology of constipation (EPOC) study in the United States: relation of clinical subtypes to sociodemographic features. Am J Gastroenterol.

[26] MacLennan AH, Taylor AW, Wilson DH, Wilson D (2000). The prevalence of pelvic floor disorders and their relationship to gender, age, parity and mode of delivery. BJOG.

