# Supplementary material for: Beyond gait and balance: urinary and bowel dysfunction in X-linked adrenoleukodystrophy
Source: Orphanet J Rare Dis. 2021 Jan 6;16:14. doi: 10.1186/s13023-020-01596-1 (PMC7789359; doi:10.1186/s13023-020-01596-1)
Supplement: Supplementary file 1 — Additional file 1. Standardized algorithm. Standardized approximations of the upper and lower bounds of the range for age of symptom onset. [file 13023_2020_1596_MOESM1_ESM.docx]

**Additional File 1.** Standardized algorithm

| **Patient-reported Value** | **Upper Bound of Range** | **Lower Bound of Range** |
| --- | --- | --- |
| 3-8 years ago* | 3 years ago | 8 years ago |
| A couple of years ago | 2 years ago | 3 years ago |
| A few years ago | 2 years ago | 4 years ago |
| Several years ago | 4 years ago | 7 years ago |
| Many years ago | 10 years ago | Youngest age reported in cohort for same symptom |
| Childhood | 12 years old | 5 years old |
| Teens | 19 years old | 13 years old |
| Early 30s** | 33 years old | 30 years old |
| Mid 30s** | 37 years old | 34 years old |
| Late 30s** | 39 years old | 37 years old |
| In my 30s** | 39 years old | 30 years old |

Standardized approximations of the upper and lower bound of the range for age of symptom onset.

*Approximation applies to all reported ranges

**Approximation applies to all decades
