# Supplementary material for: Beyond gait and balance: urinary and bowel dysfunction in X-linked adrenoleukodystrophy
Source: Orphanet J Rare Dis. 2021 Jan 6;16:14. doi: 10.1186/s13023-020-01596-1 (PMC7789359; doi:10.1186/s13023-020-01596-1)
Supplement: Supplementary file 2 — Additional file 2. Urinary and bowel symptoms in ALD/AMN. Survey distributed to all adults with ALD seen in the MGH Leukodystrophy Clinic as well as a cohort of adults with ALD not seen as patients in our clinic. [file 13023_2020_1596_MOESM2_ESM.pdf]

URINARY AND BOWEL SYMPTOMS IN ALD/AMN

We are performing a study to better understand urinary and bowel symptoms in adults with ALD/AMN. We ask that you complete this *voluntary* survey in order to help us focus our efforts on symptoms that are most important to patients. If you have any questions about this survey, please contact Camille Corre at ccorre@partners.org or (617) 724-6374. When the survey is complete, please return to the following address:

Center for Rare Neurological Diseases
Attn: Camille Corre
175 Cambridge Street, Suite 340
Boston, MA 02114

Thank you in advance for your participation!

Subject Code: _______________ **Date of Survey Completion:** __ / __ / ____

ALD Symptoms:

1. Do you experience any of the following symptoms?

Difficulty walking	☐ no	☐ yes, beginning at age ___
Difficulty with balance	☐ no	☐ yes, beginning at age ___
Leg numbness	☐ no	☐ yes, beginning at age ___
Leg weakness	☐ no	☐ yes, beginning at age ___

2. Do you use any of the following assistive devices?

Foot/leg braces	☐ no	☐ yes, beginning at age ___
Cane	☐ no	☐ yes, beginning at age ___
Crutches	☐ no	☐ yes, beginning at age ___
Manual wheelchair	☐ no	☐ yes, beginning at age ___
Power wheelchair	☐ no	☐ yes, beginning at age ___
Electric scooter	☐ no	☐ yes, beginning at age ___

3. Do you use a wheelchair or scooter full-time? ☐ no ☐ yes, beginning at age ___

4. Have you ever been diagnosed with a cerebral (brain) lesion based on an MRI?
☐ no ☐ yes, at age ___

5. Have you ever received a stem cell or bone marrow transplant as treatment for ALD? ☐ no ☐ yes, at age ___

Urinary and Bowel Symptoms:

6. **Do you feel as though you <u>urinate more frequently</u> than the average person?**
   ☐ no ☐ yes, beginning at age ___
   If yes, approximately how many times do you urinate in a 24-hour period? ____

7. **Do you ever feel a <u>sudden, urgent, compelling desire to urinate</u>, which is difficult to ignore?**
   ☐ no ☐ yes, beginning at age ___
   If yes, approximately how frequently does this sensation occur, on average?
   ☐ multiple times daily ☐ once daily
   ☐ several times a week ☐ once a week
   ☐ several times a month ☐ once a month
   ☐ less frequently than once a month
   If yes, when this sensation occurs, approximately how much time do you have to reach the restroom before you have an accident? ______________________

8. **Do you ever have <u>difficulty initiating a urine stream</u> even when you feel an urge to urinate?**
   ☐ no ☐ yes, beginning at age ___

9. **Do you ever have <u>urinary accidents</u> or involuntary leakage of urine?**
   ☐ no ☐ yes, beginning at age ___
   If yes, how often does this occur?
   ☐ multiple times daily ☐ once daily
   ☐ several times a week ☐ once a week
   ☐ several times a month ☐ once a month
   ☐ less frequently than once a month

10. **Do you ever have a sensation that you are <u>unable to completely empty your bladder</u>?**
   ☐ no ☐ yes, beginning at age ___
   If yes, how often does this occur?
   ☐ multiple times daily ☐ once daily
   ☐ several times a week ☐ once a week
   ☐ several times a month ☐ once a month
   ☐ less frequently than once a month

11. **Do you ever feel a <u>sudden, urgent, compelling desire to defecate</u>, which is difficult to ignore?**

☐ no ☐ yes, beginning at age ___

If yes, approximately how frequently does this sensation occur, on average?

☐ multiple times daily ☐ once daily
☐ several times a week ☐ once a week
☐ several times a month ☐ once a month
☐ less frequently than once a month

If yes, when this sensation occurs, approximately how much time do you have to reach the restroom before you have an accident? ______________________

12. **Do you ever have <u>bowel accidents</u> or involuntary leakage of feces?**

☐ no ☐ yes, beginning at age ___

If yes, how often does this occur?

☐ multiple times daily ☐ once daily
☐ several times a week ☐ once a week
☐ several times a month ☐ once a month
☐ less frequently than once a month

13. **Do you experience <u>constipation</u>?**

☐ no ☐ yes, beginning at age ___

If yes, how often does this occur?

☐ multiple times daily ☐ once daily
☐ several times a week ☐ once a week
☐ several times a month ☐ once a month
☐ less frequently than once a month

14. **Do you experience <u>diarrhea</u>?**

☐ no ☐ yes, beginning at age ___

If yes, how often does this occur?

☐ multiple times daily ☐ once daily
☐ several times a week ☐ once a week
☐ several times a month ☐ once a month
☐ less frequently than once a month

15. **Have you ever had a urinary tract infection (UTI)?** ☐ no ☐ yes, beginning at age ___

If yes, approximately how many UTIs have you had in the last year? ____

If yes, approximately how many UTIs have you had in your lifetime? ____

If yes, do you think your frequency of UTIs has increased in the last five years?

☐ no ☐ yes

Symptom Management:

16. Have you ever seen a urologist? ☐ no ☐ yes, beginning at age ____

If yes, what is his/her name? ________________________________

If yes, how frequently do you see him/her?

☐ only once ☐ inconsistently (< 1x/year)

☐ once a year ☐ more frequently than once a year

17. In order to manage your symptoms, have you ever tried...

☐ **…limiting your liquid intake during the day?**

If yes, was this helpful? ☐ yes ☐ no

☐ **…limiting your liquid intake at night or before bed?**

If yes, was this helpful? ☐ yes ☐ no

☐ **…limiting your food intake during the day?**

If yes, was this helpful? ☐ yes ☐ no

☐ **…limiting your food intake at night or before bed?**

If yes, was this helpful? ☐ yes ☐ no

☐ **…restricting your caffeine intake?**

If yes, was this helpful? ☐ yes ☐ no

☐ **…restricting your alcohol intake?**

If yes, was this helpful? ☐ yes ☐ no

☐ **…using a urinary catheter?**

If yes, was this helpful? ☐ yes ☐ no

If yes, please indicate type and frequency of use: ____________________

If yes, beginning at age ____

☐ **…using Depends or other protective pads to prevent leakage?**

If yes, was this helpful? ☐ yes ☐ no

☐ **…pelvic floor physical therapy?**

If yes, was this helpful? ☐ yes ☐ no

☐ **…using a bladder massage technique?**

If yes, was this helpful? ☐ yes ☐ no

☐ **…adhering to a strict bathroom schedule?**

If yes, was this helpful? ☐ yes ☐ no

18. Are there any other interventions you have found helpful for managing your symptoms? ☐ no ☐ yes: _______________________

Quality of Life

19. To what extent is your lifestyle limited because of your urinary/bowel symptoms (i.e. do you plan travel around the availability of restrooms, avoid eating/drinking in public spaces, change your work schedule to account for restroom needs, etc.)? Please circle.

No limitation   Minor limitations   Moderate limitations   Severe limitations

20. Which urinary/bowel symptom is the most bothersome to you? _______________

Medications

If you have ever tried any medications (over-the-counter or prescription) to manage your urinary and/or bowel symptoms (including but not limited to antibiotics for UTIs, probiotics, laxatives, Botox injections, etc.), please describe them below.

**Medication Name**	**Age Started Taking**	**Currently Taking?**	**Age Stopped Taking**	**What symptoms did this medication improve?**	**What side effects did you experience?**

If needed, continue on next page.

Medication Name	Age Started Taking	Currently Taking?	Age Stopped Taking	What symptoms did this medication improve?	What side effects did you experience?
